# The Rare-Earth Elements Doping of BaGdF_5_ Nanophosphors for X-ray Photodynamic Therapy

**DOI:** 10.3390/nano11123212

**Published:** 2021-11-26

**Authors:** Daria Kirsanova, Vladimir Polyakov, Vera Butova, Peter Zolotukhin, Anna Belanova, Zaira Gadzhimagomedova, Mikhail Soldatov, Ilia Pankin, Alexander Soldatov

**Affiliations:** The Smart Materials Research Institute, Southern Federal University, 344090 Rostov-on-Don, Russia; vlpolyakov@sfedu.ru (V.P.); vbutova@sfedu.ru (V.B.); pvzolotuhin@sfedu.ru (P.Z.); abelanova@sfedu.ru (A.B.); zgad@sfedu.ru (Z.G.); mikhailsoldatov@sfedu.ru (M.S.); pankin@sfedu.ru (I.P.); soldatov@sfedu.ru (A.S.)

**Keywords:** X-ray photodynamic therapy, cancer treatment, nanoparticles, nanophosphors, rare-earth elements

## Abstract

It is known that the initiation of photodynamic therapy (PDT) in deep-seated tumors requires the use of X-rays to activate the reactive oxygen species generation in deep tissues. The aim of this paper is to synthesize X-ray nanophosphors and analyze their structural and luminescence characteristics to push the PDT process deep into the body. The article deals with BaGdF_5_:Eu^3+^, BaGdF_5_:Sm^3+^, and BaGdF_5_:Tb^3+^ nanophosphors synthesized using microwave synthesis. It is found that the nanoparticles are biocompatible and have sizes 5–17 nm. However, according to the analysis of X-ray excited optical luminescence, BaGdF_5_:Sm^3+^ nanophosphors will not be effective for treating deep-seated tumors. Thus, BaGdF_5_:Eu^3+^ and BaGdF_5_:Tb^3+^ nanoparticles meet the requirements for the subsequent production of nanocomposites based on them that can be used in X-ray photodynamic therapy.

## 1. Introduction

Nowadays, cancer is one of the largest health problems and is the second leading cause of death worldwide. According to the GLOBOCAN 2018 database compiled by the International Agency for Research on Cancer (IARC), 18.1 million new cancer cases and 9.6 million cancer deaths occurred globally in 2018 [1]. Therefore, many procedures and drugs for cancer treatment have been developed, and many more are still being studied. For example, today, surgery, radiotherapy, and such drug treatments as chemotherapy and targeted therapy are well known. The major disadvantage of these approaches is the ability to cause serious side effects and oncologic complications during treatment.

Thus, it became necessary to develop an alternative cancer treatment called photodynamic therapy (PDT) that is both non-invasive and minimally toxic. This therapy is based on an interaction between three essential components such as a photosensitive substance (photosensitizer, PS), light activation, and molecular oxygen. The mechanism of PDT consists of two steps: The first step is the injection of PS into the patient’s body followed by its selective accumulation in the tumor tissues. At the second step, PS is activated upon irradiation with visible or near-infrared light of a specific wavelength. This process leads to the generation of reactive oxygen species (ROS) which cause harmful effects on the cancer cells. ROS are a group of highly reactive oxygen-containing chemical molecules, for example, superoxide anion (^•^O_2_^−^), hydrogen peroxide (H_2_O_2_) and hydroxyl radical (^•^OH), and singlet oxygen (^1^O_2_) [2,3]. The high ROS level inside the tumor microenvironment can break the antioxidative–oxidative balance, inhibit the growth of the tumor, and induce the apoptosis of tumor cells due to the DNA damage [4,5]. Moreover, it has been suggested that not only singlet oxygen ^1^O_2_ but also other free radicals may also play a key role as cytotoxic agents in the photodynamic damage of cancer cells [6]. Therefore, the use of PDT allows non-invasive tumor destruction while sparing healthy surrounding tissue with minimal complications during treatment. Unfortunately, this approach is ineffective for the treatment of deep-seated tumors due to some limitations. The main limitation is the shallow tissue penetration (<1 cm) of the optical radiation used in PDT. Thus, only surface tumors (on the skin or on the mucous membrane) can be treated through conventional PDT.

However, X-ray can be used as a radiation source to increase the penetration depth of light and activate the PDT process in deep-located tumor tissues [7,8]. This so-called X-ray photodynamic therapy (XPDT) is based on the use of X-ray nanophosphors that under X-ray radiation emit light in the visible range. After this, a conjugated photosensitizer is activated through Förster resonance energy transfer (FRET) followed by the generation of ROS in tumors of internal organs.

Generally, nanoparticles of luminescent materials doped with rare-earth ions are proposed as materials promising for use in XPDT [9,10,11,12,13]. For instance, nanoparticles of rare-earth fluorides (such as BaGdF_5_) are the most interesting and well-studied host materials for doping with rare-earth ions (Eu^3+^, Tb^3+^, etc.). The main advantages of the group of BaGdF_5_ nanoparticles are the low energy of emitted phonon, the possibility of multicolor tunable luminescence, as well as high resistance to X-ray and photochemical degradation [14]. The most important reason for the use of rare-earth fluorides is the possibility of restructuring the luminescence characteristics, which can be achieved by varying the concentration of the doping rare-earth ion [15,16,17,18,19]. These materials are capable to effectively convert ionizing radiation into the visible or ultraviolet regions due to a stepwise multiphoton process that occurs in the system of energy levels of lanthanide dopant ions [20,21]. The high stability of Ln^3+^ (lanthanide ions) in BaGdF_5_ nanoparticles makes them suitable for use in biological tissue cells [22,23,24], including human erythrocytes [25]. Some researchers believe that nanoparticles based on NaGdF_4_:Eu^3+^ are more promising for X-ray photodynamic therapy [10], although according to some other sources [25] during a hydrothermal synthesis NaGdF_4_ nanoparticles agglomerate to the spherical objects with an average size up to 150 nm, which is rather large for preparing the desired nanocomposite for X-ray photodynamic therapy, while BaGdF_5_ based nanoparticles have averaged size about 10 nm only. By varying the synthesis parameters, it is possible to tune the size of the resulting nanoparticles [19,26]. In most cases, the synthesis of BaGdF_5_-based nanoparticles was performed by using a standard hydrothermal approach, which is a rather stable but relatively long procedure. 

In this work, we present BaGdF_5_ X-ray nanophosphors doped with such rare-earth ions Eu^3+^, Tb^3+^, and Sm^3+^ that were synthesized with a new microwave synthesis. Besides, the structural and X-ray excited optical luminescence (XEOL) characteristics of nanoparticles and their cytotoxicity have been discussed in detail.

## 2. Materials and Methods

### 2.1. Materials

Initial precursors GdCl_3_, EuCl_3_, TbCl_3_·6H_2_O, SmCl_3_, ethylene glycol, BaCl_2_∙2H_2_O, polyethylene glycol (PEG, M = 1500 g/mol), and NH_4_F were purchased from Sigma-Aldrich Co. (St Louis, MO, USA).

### 2.2. Synthesis

Based on the solvothermal method that was adapted from work by Sudheendra et al. [27], we developed a new microwave synthesis that was reported for the first time in our previous work [28]. This method was used to synthesize BaGdF_5_:Eu^3+^, BaGdF_5_:Sm^3+^, and BaGdF_5_:Tb^3+^ nanophosphors.

The preparation of BaGdF_5_:Ln^3+^ (Ln^3+^ = Eu^3+^, Sm^3+^, Tb^3+^) using microwave synthesis was carried out as follows. At the first stage, 0.9 mmol (237.2 mg) GdCl_3_ and 0.1 mmol LnCl_3_ (or LnCl_3_·6H_2_O) were dissolved in 20 mL of ethylene glycol under ultrasonic treatment for 10 min. Then, 1 mmol (244.2 mg) BaCl_2_∙2H_2_O was added to the solution and mixed for 30 min followed by the addition of 1.5 g PEG and subsequent ultrasonic treatment for 15 min. At the next stage, in a separate vessel, 5.5 mmol (203.7 mg) NH_4_F was dissolved in 10 mL of ethylene glycol, mixed, and suspended in an ultrasonic bath for 30 min. The obtained suspension was transferred to a Teflon ampoule and placed in a microwave oven (Mars6, CEM Corporation, Matthews, NC, USA). The reaction mixture was heated up to 200 °C for 20 min and then kept at this temperature for 2 h while the power of the microwave reactor was 600 W. After that, the ampoule was cooled down to room temperature and the precipitate was washed 3 times with distilled water using centrifugation (11,000 rpm for 20 min) which was then followed by drying at 60 °C in a vacuum chamber overnight. The resulting samples were marked as BaGdF_5_:Eu, BaGdF_5_:Tb, and BaGdF_5_:Sm. 

### 2.3. Characterization

The X-ray diffraction (XRD) of the synthesized nanoparticles was measured by the Bruker D2 PHASER X-ray diffractometer (Bruker AXS Inc., Fitchburg, WI, USA) using Cu Kα radiation (*λ* = 1.5406 Å) at 30 kV and 10 mA. For the measurements, we used a low-background cuvette and the following conditions: 2θ range—5°–90°, step size—0.01°. Distances between atomic planes were analyzed with high-resolution transmission electron microscopy (HRTEM) using the FEI Tecnai G2 F20 (FEI, Hillsboro, OR, USA) microscope. The shape and size of the particles were studied with transmission electron microscopy (TEM) using the Tecnai G2 Spirit TWIN microscope (FEI, Hillsboro, OR, USA). The potential stability of the colloidal system was analyzed with zeta potential using stability analysis system Stabino (Particle Metrix GmbH, Inning am Ammersee, Germany). The elemental composition was analyzed using micro-X-ray fluorescence spectrometer M4 TORNADO (Bruker, Billerica, MA, USA). IR spectra were measured on a Bruker Vertex 70 spectrometer (Bruker AXS Inc., Fitchburg, WI, USA) in ATR geometry (Attenuated total reflectance) using an MCT detector and a Bruker Platinum ATR attachment. The spectra were measured in the range from 5000–500 cm^−1^ with a resolution of 1 cm^−1^ and 128 scans. The reference was air. Nitrogen adsorption–desorption isotherms were measured at −196 °C obtained on Accelerated Surface Area and Porosimetry analyzer ASAP 2020 (Micromeritics Instruments Corp., Norcross, GA USA). The samples were activated at 250 °C for 10 h under a dynamic vacuum before the measurement. X-ray-excited optical luminescence (XEOL) signal was detected by using Agilent Cary Eclipse fluorescence spectrophotometer with emission slit set to 10 nm and following parameters of X-ray tube: voltage 35 kV and current 1.6 mA. Powder samples were deposited on the thin film which was fixed in a way that result in an angle of 45° between the sample surface and both X-ray beam and fluorescence detector window.

### 2.4. Cytotoxicity and ROS Generation Assays

In this study, HeLa and K562 cell lines were used as the in vitro experimental models for cytotoxicity testing and analyzing ROS-modulating effects of the synthesized nanomaterials. Hela and K562 cells were kind gifts from Southern Centre of Russian Academy of Science (Rostov-on-Don, Russia) and Rostov-on-Don Oncology Institute (Rostov-on-Don, Russia), respectively. The cells were grown in 24-well plates (SPL Lifesciences, Pocheon, South Korea) in GlutaMax DMEM medium (Thermo Fisher Scientific, Waltham, MA, USA) supplemented with 10% of fetal bovine serum (GE Healthcare, Chalfont St Giles, UK), 50 IU/mL of penicillin, and 50 µg/mL of streptomycin (Thermo Fisher Scientific, Waltham, MA, USA). The cells were kept at 37 °C and 5% CO_2_ in the Sanyo MCO-18AC incubator (Panasonic, Osaka, Japan). Cell growth was controlled using the Premiere MIS-9000 inverted microscope (C&A, Shanghai, China).

To analyze the cytotoxicity of the materials, a trypan blue exclusion assay was performed on HeLa cells using the automated cell viability analyzer Countess II FL according to the manufacturer’s protocol (Thermo Fisher Scientific, Waltham, MA, USA). During the experiment, stock solutions of nanomaterials in saline were introduced into the culture medium at the concentration of 50 μg/mL. In the control group, saline was added to the medium. Following adding the test samples, the cells were incubated for 24 h.

For assessing ROS-modulating effects, flow cytometry was performed on K562 cells exposed to 50 μg/mL nanomaterials or vehicle for 1 h. This analysis was carried out on the CytoFlex flow cytometer (Beckman Coulter, Brea, CA, USA) using the following molecular probes:

- CellROX Green (CRG)—a probe for mitochondrial and nuclear ROS;
- CellROX Orange (CRO)—a probe for cytosolic ROS;
- 7-AAD—a control dye for gating viable cells.

All dyes were used in accordance with the manufacturer’s recommendations (Thermo Fisher Scientific, Waltham, MA, USA). At least 10,000 events were analyzed in each sample. Only viable singlets were analyzed. Signals from the molecular probes were normalized to the FS channel. This algorithm allows increasing analytical sensitivity and specificity of the method, as well as compensating the differences between the signal levels and the actual functional parameters of cells varying in dimensions.

## 3. Results and Discussion

### 3.1. X-ray Diffraction

**Figure 1:**
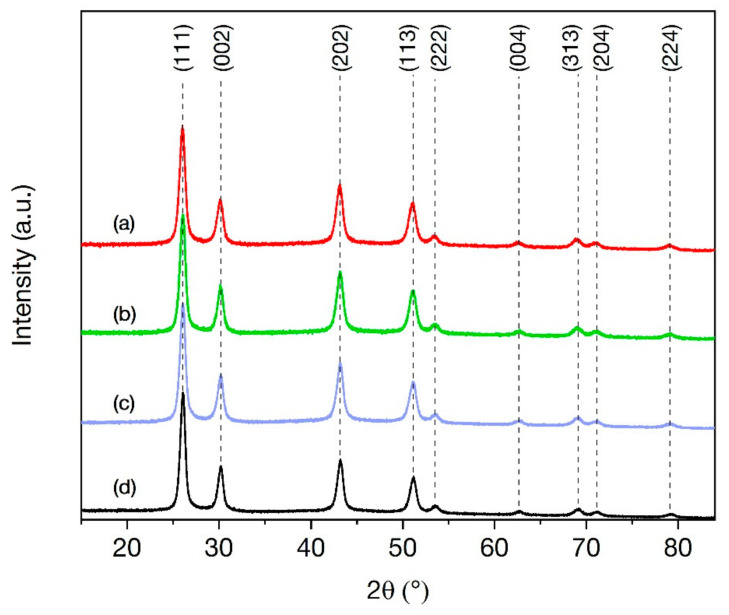
XRD patterns of the synthesized samples (**a**) BaGdF_5_:Eu, (**b**) BaGdF_5_:Tb, and (**c**) BaGdF_5_:Sm. For comparison, the (**d**) BaGdF_5_ profile is presented.

The *Jana2006* program package (Version 25 October 2015; Academy of Sciences, Institute of Physics, Praha, Czech Republic) was used for profile analysis [29]. It was found that all synthesized samples are single-phase materials with a cubic phase of the *Fm*-3*m* (225) space group, as stated in the literature data (JCPDS card no. 24-0098 [30]) (Figure 1). The refined value of the cell parameters of BaGdF_5_:Eu, BaGdF_5_:Tb, and BaGdF_5_:Sm samples are 5.9314(3) Å, 5.9279(3) Å, and 5.9265(3) Å (cell volumes: 208.676(18) Å^3^, 208.302(19) Å^3^, and 208.161(17) Å^3^), respectively.

The BaGdF_5_ crystal structure is formed from the BaF_2_ lattice (space group *Fm*-3*m*, a = 6.2001 Å [31]) by replacing Ba^2+^ ions with Gd^3+^ ions (ionic radii are 1.49 Å and 1.078 Å, respectively [32]). During the replacement, the decrease in cell parameters is observed. This fact can be seen from the profile analysis. Moreover, it is found that different doped rare-earth ions lead to slightly different cell parameters of our samples. This phenomenon can be explained by the various amounts of F^−^ anions. The uncompensated positive charge of the trivalent ion leads to the inclusion of additional fluorine ions in the lattice. Note that some F^−^ ions could displace from the anion site and cause structure relaxation and distortion [33]. The mutual repulsion of F^−^ ions leads to an increase of cell parameters. This fact is consistent with XRF analysis, for example, the BaGdF_5_:Eu structure has the largest cell parameter according to the biggest amount of F^−^ in the sample (see Section 3.2).

Furthermore, the average crystalline size was estimated using the Debye–Scherrer equation. The analysis of the full width at half-maximum (FWHM) of XRD lines shows that for the synthesized BaGdF_5_:Eu, BaGdF_5_:Tb, and BaGdF_5_:Sm nanoparticles the averaged sizes equal to 9.56, 9.91, and 9.55 nm, respectively. These data are consistent with TEM analysis (see Section 3.4).

### 3.2. X-ray Fluorescence (XRF)

The elemental composition of the synthesized materials determined by XRF analysis are presented in Table 1.

As can be seen, the doping elements are not uniformly included in the crystal lattice of BaGdF_5_. It is known that the efficiency of isomorphic replacement largely depends on the ionic radii of the doping elements. Therefore, it is expected that lanthanides with an ionic radius closest to the Gd^3+^ ionic radius will most effectively replace it in the crystal lattice. We observe this trend by analyzing the results of the elemental composition. The ionic radii of hexacoordinated Sm^3+^, Eu^3+^, Gd^3+^, and Tb^3+^ are 1.098, 1.087, 1.078, and 1.063 Å, respectively [32]. Sm^3+^ ion, as the largest, is the worst embedded in the lattice. Thus, the molar ratio of elements in the BaGdF_5_:Eu, BaGdF_5_:Sm, and BaGdF_5_:Tb was 1:1.28:0.12, 1:1.13:0.08, and 1:1.18:0.12, respectively, which is close to the initial molar ratio of precursors 1:0.9:0.1.

### 3.3. High-Resolution TEM (HRTEM)

**Figure 2:**
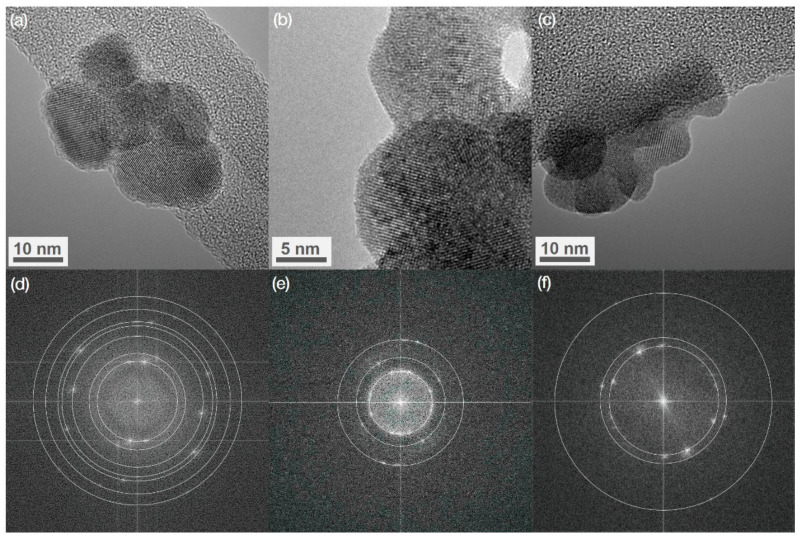
HRTEM images of (**a**) BaGdF_5_:Eu, (**b**) BaGdF_5_:Tb, and (**c**) BaGdF_5_:Sm; (**d**–**f**) FFT images with circles marking the spacing between atomic planes.

Using the *ImageJ* program (Version 1.52p, Wayne Rasband (National Institute of Health), Bethesda, MD, USA), the two-dimensional fast Fourier transformation (FFT) was performed for HRTEM images (Figure 2). The bottom part of Figure 2 presents FFT images with circles marking the spacing between atomic planes. In this figure, the weak diffraction spots are related to the diffraction on different planes. The distances between atomic planes were analyzed using circular selection. The interplanar distances in BaGdF_5_:Eu were approximately 0.34, 0.30, 0.21, 0.18, 0.17, and 0.15 nm, which corresponds to the distances (111), (002), (202), (113), (222), and (004) planes, respectively. For BaGdF_5_:Tb, it was observed that d-spacing are about 0.30, 0.21, and 0.15 nm, corresponding to (002), (202), and (004) planes, respectively. For BaGdF_5_:Sm, d-spacing of 0.34, 0.30, and 0.17 nm could be indexed as (111), (002), and (222) planes, respectively.

### 3.4. Transmission Electron Microscopy (TEM) 

**Figure 3:**
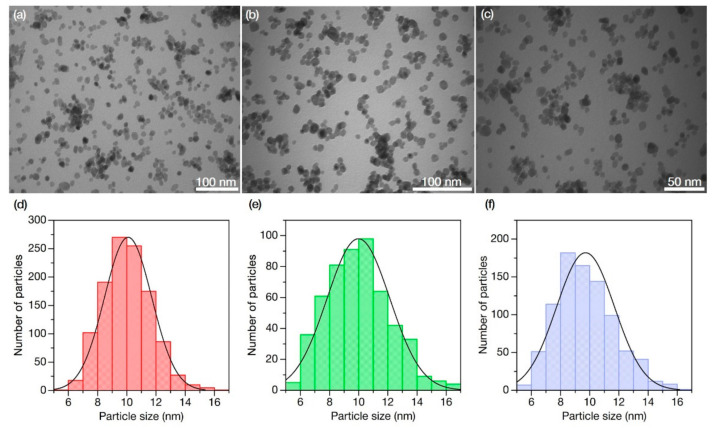
TEM images of (**a**) BaGdF_5_:Eu, (**b**) BaGdF_5_:Tb, and (**c**) BaGdF_5_:Sm; Particle size distribution of (**d**) BaGdF_5_:Eu, (**e**) BaGdF_5_:Tb, and (**f**) BaGdF_5_:Sm according to TEM analysis.

The size distribution of nanoparticles was estimated using the *ImageJ* program [34] analyzing TEM images (Figure 3). Total numbers of measured nanoparticles were 1140, 530, and 879 for BaGdF_5_:Eu, BaGdF_5_:Tb, and BaGdF_5_:Sm, respectively. As a result, it has been shown that for all samples nanoparticles are in the range of 5–17 nm with predominant fractions of ~10 nm. This fact complies with the requirements for nanophosphors as a part of nanocomposites that can be used in XPDT [35,36]. Thus, according to the obtained particle size distribution, all synthesized nanomaterials are suitable for the subsequent synthesis of nanocomposites for XPDT.

### 3.5. Zeta-Potential

The potential stability of the colloidal system was estimated with the magnitude of the zeta potential. It was found out that all three samples have a positively charged surface. However, sample BaGdF_5_:Eu showed higher zeta-potential (32.4 mV) in comparison with BaGdF_5_:Tb (27.7 mV) and BaGdF_5_:Sm (23.6 mV) in distilled water, and thus these nanoparticles are more stable.

### 3.6. Nitrogen Adsorption

**Figure 4:**
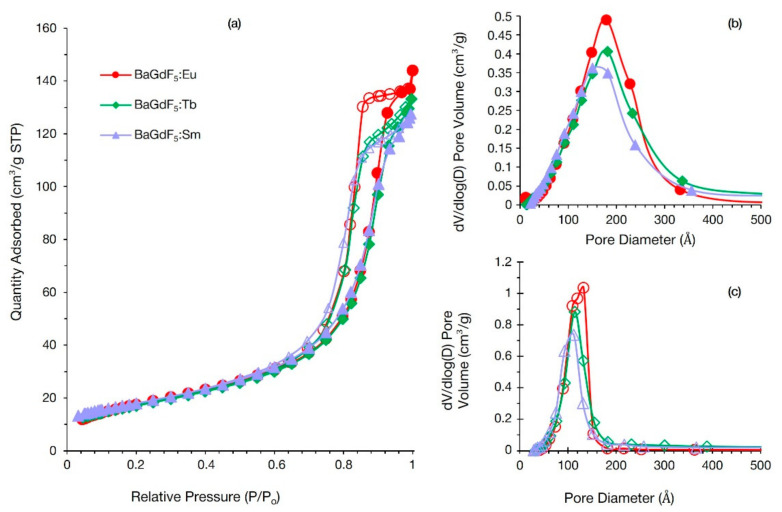
(**a**) Nitrogen sorption isotherms of synthesized samples BaGdF_5_:Eu (circle markers), BaGdF_5_:Tb (diamond markers), and BaGdF_5_:Sm (triangle markers). Adsorption branches of isotherms pointed with filled markers, while desorption ones—empty markers. BJH adsorption pore distribution obtained from the N_2_ adsorption isotherms (**b**). Panel (**c**) as panel (**b**) from the desorption isotherms.

Nitrogen sorption isotherms for all obtained samples are provided in Figure 4a. All samples are exhibiting similar sorption profiles, and the isotherms can be attributed to type III (IUPAC classification), which is typical for non-porous or macroporous materials. In the high-pressure region, all isotherms show pronounced hysteresis loops. They could be attributed to type H1 associated with capillary condensation of nitrogen in spaces between uniform nanoparticles in agglomerates. Specific surface areas calculated by the Brunauer–Emmett–Teller (BET) model were estimated as 64, 61, and 64 m^2^/g for BaGdF_5_:Eu, BaGdF_5_:Tb, and BaGdF_5_:Sm, respectively. The consequent Barrett–Joyner–Halenda (BJH) pore size distribution is shown in Figure 4b,c as determined from adsorption and desorption isotherms, respectively. Narrow peaks indicate uniform slit-like cages of ~10 nm between nanoparticles in agglomerates in good agreement with TEM data.

### 3.7. Fourier Transform Infrared (FTIR) Spectroscopy

**Figure 5:**
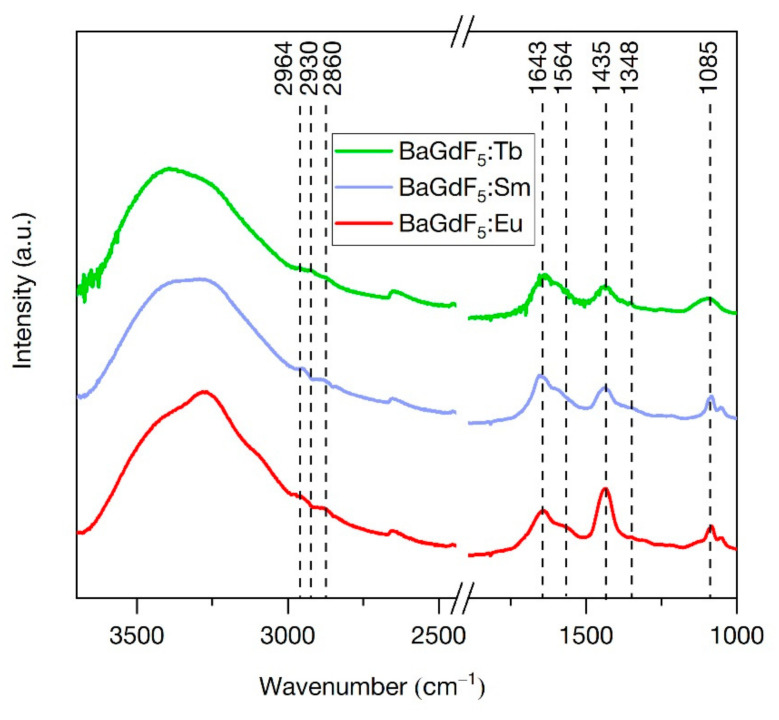
FTIR spectra of the synthesized samples.

The purity of the final products was monitored by FTIR spectroscopy (Figure 5). All samples have a similar IR profile. The broad peak at 3500–3000 cm^−1^, as well as at 1643 cm^−1^, corresponds to the stretching and bending vibrations of water molecules adsorbed on the surface of the nanoparticles. The peak at 1435 cm^−1^ corresponds to the stretching vibrations of the Gd-F and Ba-F bonds (NIST Chemistry WebBook, CAS Registry Number: 7787-32-8, Access date: 30 September 2021) [37]. The peaks at 1050 and 1085 cm^−1^ correspond to the O-H bonds of primary alcohol groups and the ether bonds of PEG chains, respectively [38]. We can also observe a low-intensity peak at 2964 cm^−1^, associated with vibrations of the isopropanol methyl groups, which was used to wash the device before measurements, and two low-intensity peaks at 2930 and 2860 cm^−1^, corresponding to asymmetric and symmetric CH_2_ stretching, respectively. Thus, it can be concluded that PEG molecules are present on the surface of nanoparticles. All results are consistent with literature data [39,40,41].

### 3.8. X-ray Excited Optical Luminescence (XEOL)

**Figure 6:**
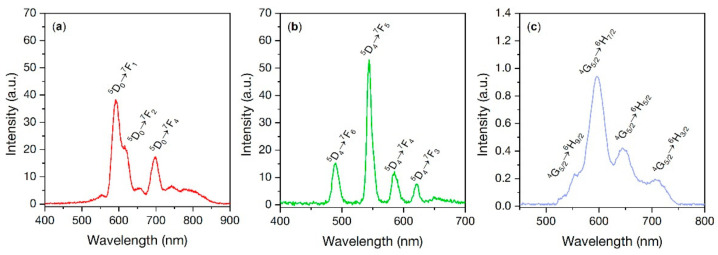
XEOL spectra measured for (**a**) BaGdF_5_:Eu, (**b**) BaGdF_5_:Tb, (**c**) BaGdF_5_:Sm. The numbered peaks correspond to ^5^D_0_ → ^7^F_1_, ^5^D_0_ → ^7^F_2_, ^5^D_0_ → ^7^F_4_ transitions for BaGdF_5_:Eu; ^5^D_4_ → ^7^F_6_, ^5^D_4_ → ^7^F_5_, ^5^D_4_ → ^7^F_4_, ^5^D_4_ → ^7^F_3_ transitions for BaGdF_5_:Tb; and ^4^G_5/2_ → ^6^H_9/2_, ^4^G_5/2_ → ^6^H_7/2_, ^4^G_5/2_ → ^6^H_5/2_, ^4^G_5/2_ → ^6^H_3/2_ transitions for BaGdF_5_:Sm, respectively.

Rare-earth ions such as Eu^3+^, Tb^3+^, and Sm^3+^ doped into a wide range of materials exhibit optical luminescence. On the one hand, the latter makes it possible to convert the ionizing radiation into visible light as a part of the XPDT system. On the other hand, a study of certain intensities of transitions in XEOL spectra could give additional information about the environment of the lanthanide ion. XEOL spectra for all synthesized samples are depicted in Figure 6. The BaGdF_5_:Eu XEOL spectrum shows spectral shape a typical for Eu^3+^ ion doped into BaGdF_5_ matrix. The origin of Eu^3+^ emission spectrum in the optical range is forbidden electric-dipole 4f → 4f (^5^D_0_ → ^7^F_J=0,1,2,3,4_) transitions which strongly depend on the symmetry of the site which Eu^3+^ occupies [42]. While magnetic-dipole allowed ^5^D_0_ → ^7^F_1_ (*λ* = 592 nm) and ^5^D_0_ → ^7^F_4_ (*λ* = 698 nm) transitions are relatively insensitive to the environment, low intensity of electric-dipole transition ^5^D_0_ → ^7^F_2_ (*λ* = 618 nm) as well as the absence ^5^D_0_ → ^7^F_0_ transition could be a signature of high symmetry at the Eu^3+^ site. As for BaGdF_5_:Tb, it is characterized by four strong narrow bands that correspond to Tb^3+^ transitions ^5^D_4_ → ^7^F_6_ (*λ* = 490 nm), ^5^D_4_ → ^7^F_5_ (*λ* = 545 nm), ^5^D_4_ → ^7^F_4_ (*λ* = 585 nm), and ^5^D_3_ → ^7^F_6_ (*λ* = 621 nm). While four peak observed for BaGdF5:Sm can be associated with following Sm^3+^ transitions ^4^G_5/2_ → ^6^H_9/2_ (*λ* = 554 nm), ^4^G_5/2_ → ^6^H_7/2_ (*λ* = 596 nm), ^4^G_5/2_ → ^6^H_5/2_ (*λ* = 646 nm), and ^4^G_5/2_ → ^6^H_3/2_ (*λ* = 708 nm) [43]. 

However, the intensity of the luminescence from this sample of Sm-doped is substantially less compared to that from BaGdF_5_:Eu and BaGdF_5_:Tb. Thus, Gaedtke et al. [44] showed that the XEOL intensity of LaF_3_-type materials increases with an increase of the Sm^3+^ concentration up to 5%, while with further increase of Sm^3+^ concentration it tends to decrease. At Sm^3+^ concentration of more than 10%, the luminescence practically does not appear and complete quenching occurs. The quenching of XEOL can be caused by various factors, such as an optical scattering increase with an increase of the Sm^3+^ concentration, as well as an increase in the number of non-radiative recombination sites located on the surface of the nanoparticles. However, even the authors question the influence of these two factors. Thus, the reason for the quenching of XEOL at Sm^3+^ content of 10% or more remains unclear.

### 3.9. Cytotoxicity Analysis

**Figure 7:**
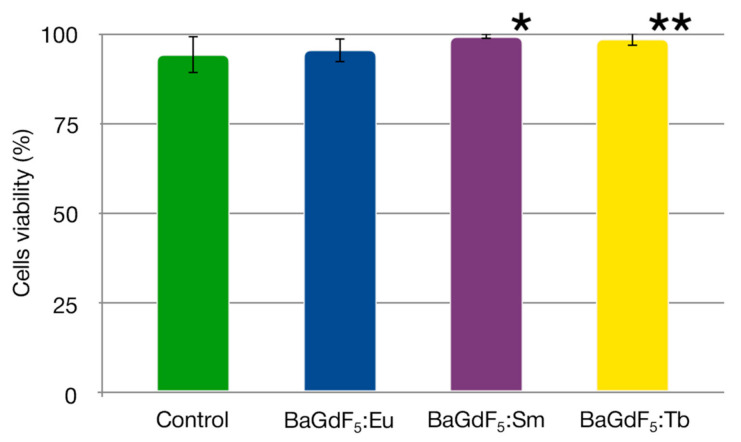
Viability of HeLa cells (%) in the control group and after exposure to synthesized nanoagents with the final concentration of 50 μg/mL for 24 h. The error bars correspond to the standard deviation. *—*p* = 0.011, **—*p* = 0.051 (compared to the control group).

The toxicity of BaGdF_5_-based nanomaterials doped with different rear-earth ions was evaluated using the trypan blue exclusion assay on HeLa cells. It was found that different formulations, at the same concentration of 50 μg/mL, significantly differed in cytotoxicity (Figure 7).

Under the tested conditions, BaGdF_5_:Eu demonstrated no prominent toxicity towards HeLa cells. Surprisingly, BaGdF_5_:Sm and BaGdF_5_:Tb formulations increased the viability of the cells by 5.3% (*p* = 0.011) and 4.5% (*p* = 0.051), respectively. 

### 3.10. ROS Generation (In Vitro)

For potential XPDT application, the scintillating nanoparticles must be conjugated with photosensitizer molecules, which are invoked for efficient ROS generation when such composite materials are exposed by ionizing radiation. However, the impact of the ScNPs themselves on the oxidative status of the cells is also important upon choosing the best candidates for further composite construction.

**Figure 8:**
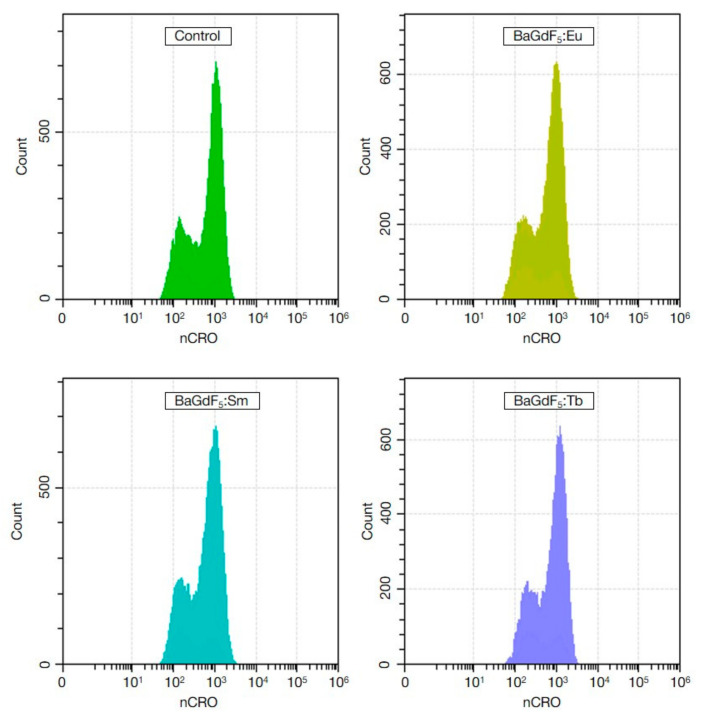
The histograms of cell distribution by nCRO+ signal intensity (ROS cytosol levels normalized by cell size) of K562 cells in the control group and after exposure to synthesized nanoagents with the final concentration of 50 μg/mL for 1 h.

**Figure 9:**
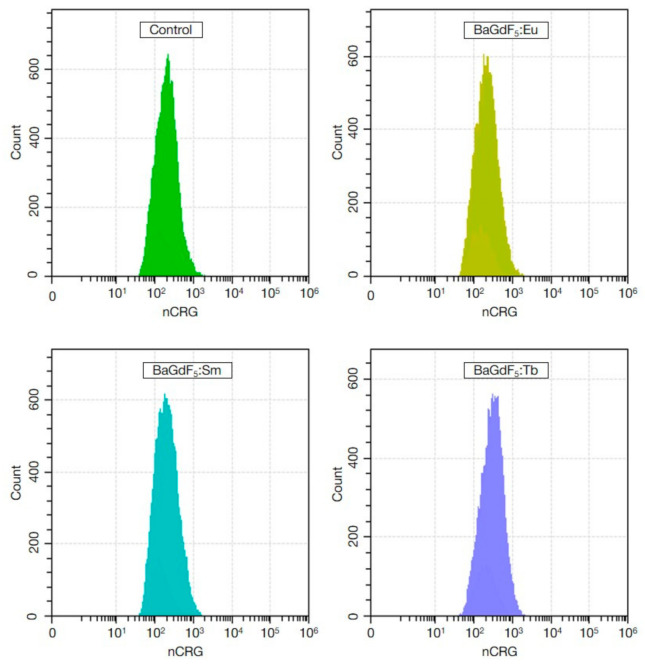
The histograms of cell distribution by nCRG+ signal intensity (ROS levels of mitochondria and nuclei normalized by cell size) of K562 cells in the control group and after exposure to synthesized nanoagents with the final concentration of 50 μg/mL for 1 h.

To assess the ROS-inducing capabilities of the nanoformulations, the flow cytometry coupled with cytosolic and mitochondrial/nuclear ROS probes was used (Figure 8 and Figure 9).

It can be seen on the signal vs. events count histograms that no apparent qualitative changes were observed in both CRO and CRG channels.

Quantitative analysis showed that the studied groups significantly differed in oxidative status, with the most notable changes in nuclear and mitochondrial ROS generation following the nanoformulations treatment (Table 2).

**Figure 10:**
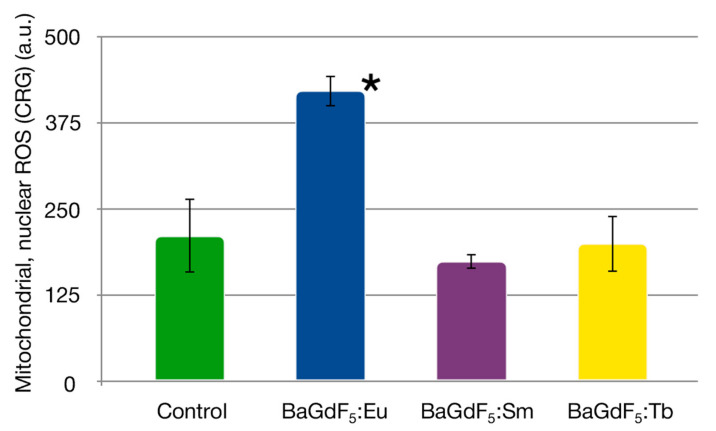
CRG signal intensity (mitochondrial and nuclear ROS levels) in K562 cells following 1 h exposure to the nanoagents at 50 μg/mL and in the control group. *—*p* < 0.001.

It can be seen from the data that BaGdF_5_:Eu caused a significant, twofold, increase in mitochondrial/nuclear ROS (Figure 10).

Summarizing the biocompatibility assays results, all three tested nanophosphors were non-toxic under the studied conditions. Notably, BaGdF_5_:Sm and BaGdF_5_:Tb nanoparticles demonstrated cell viability-promoting effects. These effects were probably modulated by induction of cytoprotective pathways, but no significant changes were observed in gross biochemical oxidative status parameters, i.e., the formulations did not cause oxidative stress, unlike the BaGdF_5_:Eu formulation, which promoted profound mitochondrial/nuclear ROS generation without significantly affecting the viability of the cells. However, as only the first wave of ROS generation was assessed (the one developing within one-hour post-treatment with redox-active agents), and viability was only assessed after one cell cycle, we plan to further test whether there are any delayed oxidative stress-related consequences of BaGdF_5_:Eu exposure. Redox-modulating properties of BaGdF_5_:Eu are interesting due to practical considerations. Nuclear protective systems of the cell respond to pro-oxidative shifts less effectively than to the cytosolic ones, and BaGdF_5_:Eu demonstrates unique properties towards balancing ROS in the cell. Such agents are known to effectively damage DNA and cause cell death by various mechanisms (from metabolic catastrophe to necrosis and autoschizis), and thus detailed data on the redox mechanisms of the nanoformulations will be collected and analyzed in further experiments. 

## 4. Conclusions

The developed microwave synthesis of X-ray nanophosphors based on BaGdF_5_ doped with rare-earth ions made it possible to obtain nanoparticles 5–17 nm in size with a significant decrease (at least an order of magnitude) in the synthesis time compared to the traditional solvothermal method. According to their dimensional characteristics, these nanoparticles meet the requirements for the subsequent production of nanocomposites based on them that can be used in X-ray photodynamic therapy.

According to XEOL analysis, BaGdF_5_:Sm demonstrated substantially lower luminescence intensity compared with Tb- and Eu-doped NPs. This finding revealed less efficient energy transfer between matrix and luminescent sites in Sm-doped NPs or more pronounced luminescence quenching, thus making it potentially less efficient for XPDT application.

Using cytotoxicity testing and the flow cytometry cellular ROS assay, it was demonstrated that the three nanoformulations were generally non-toxic at the tested concentration within one cell cycle (with BaGdF_5_:Sm and BaGdF_5_:Tb even promoting cells survival). BaGdF_5_:Eu featured fast (within 1 h) twofold induction of nuclear and mitochondrial ROS. Apparently, the three nanoagents have complex biochemical and signaling effects leading to rearrangement of signaling circuitry of the cell manifesting in survival modulation and compartment-specific ROS generation.

## Figures and Tables

**Table 1:** Elemental composition of the samples measured by XRF.

Sample	Elemental Composition (at.%) by XRF					
	Ba	Gd	Eu	Tb	Sm	F
BaGdF_5_:Eu	11.64	14.9	1.37	-	-	72.09
BaGdF_5_:Tb	12.13	14.39	-	1.51	-	71.97
BaGdF_5_:Sm	12.75	14.41	-	-	1.02	71.81

**Table 2:** CRO and CRG signals intensity in the studied cells after exposure to 50 μg/mL of nanoagents for 1 h and in the control group.

Group	Analyzed Parameter			
	CRO Channel		CRG Channel	
	M ± SD ^1^, a.u.	pMW ^2^	M ± SD, a.u.	pMW
Control	578.70 ± 114,77	-	211.28 ± 53,93	-
BaGdF_5_:Eu	507.60 ± 14,77	0.353	421.75 ± 22.31	<0.001
BaGdF_5_:Tb	658.55 ± 98.45	0.212	199.78 ± 40.77	1.000
BaGdF_5_:Sm	638.64 ± 36.31	0.402	174.13 ± 10.65	0.212

^1^ Mean and standard deviation. ^2^ Significance level according to the Mann–Whitney U test.

## Data Availability

Data sharing is not applicable to this article.
